# Optimizing graph-based patterns to extract biomedical events from the literature

**DOI:** 10.1186/1471-2105-16-S16-S2

**Published:** 2015-10-30

**Authors:** Haibin Liu, Karin Verspoor, Donald C Comeau, Andrew D MacKinlay, W John Wilbur

**Affiliations:** 1 NCBI, 8600 Rockville Pike, 20894 Bethesda, MD, USA; 2 NICTA, Lvl 2 / Bldg 193, The University of Melbourne, 3010 VIC, Australia; 3 Department of Computing and Information Systems, The University of Melbourne, 3010 VIC, Australia

**Keywords:** biomedical event extraction, approximate subgraph matching, graph-based pattern optimization

## Abstract

**In BioNLP-ST 2013:**

We participated in the BioNLP 2013 shared tasks on event extraction. Our extraction method is based on the search for an approximate subgraph isomorphism between key context dependencies of events and graphs of input sentences. Our system was able to address both the GENIA (GE) task focusing on 13 molecular biology related event types and the Cancer Genetics (CG) task targeting a challenging group of 40 cancer biology related event types with varying arguments concerning 18 kinds of biological entities. In addition to adapting our system to the two tasks, we also attempted to integrate semantics into the graph matching scheme using a distributional similarity model for more events, and evaluated the event extraction impact of using paths of all possible lengths as key context dependencies beyond using only the shortest paths in our system. We achieved a 46.38% F-score in the CG task (ranking 3*^rd^*) and a 48.93% F-score in the GE task (ranking 4*^th^*).

**After BioNLP-ST 2013:**

We explored three ways to further extend our event extraction system in our previously published work: (1) We allow non-essential nodes to be skipped, and incorporated a node skipping penalty into the subgraph distance function of our approximate subgraph matching algorithm. (2) Instead of assigning a unified subgraph distance threshold to all patterns of an event type, we learned a customized threshold for each pattern. (3) We implemented the well-known Empirical Risk Minimization (ERM) principle to optimize the event pattern set by balancing prediction errors on training data against regularization. When evaluated on the official GE task test data, these extensions help to improve the extraction precision from 62% to 65%. However, the overall F-score stays equivalent to the previous performance due to a 1% drop in recall.

## Introduction

Identifying biomedical events is of significant importance to the understanding of sophisticated interactions between physiological processes and disease and their comprehensive downstream effects on the behavior of biomedical systems at a systems biology level. As a community-wide competition, the BioNLP shared task series has led to a noticeable development of text mining resources and techniques for the automated extraction of semantic events from the biomedical literature such as protein binding, DNA methylation and regulatory events [1,2]. An event describes the interaction among multiple participants with diverse semantic roles [3]. Biomedical events usually have a complex internal structure and can be divided into a set of nested events. Capturing such causal event structures is necessary for the automatic reconstruction of detailed biological pathways.

While BioNLP-ST 2009 and 2011 focused on molecular and sub-cellular level events, BioNLP-ST 2013 extended the scope to biological processes at higher levels of organization by introducing many new biological issues such as organ growth, blood vessel development, pathway curation and cancer genetics. As a team from NCBI (National Center for Biotechnology Information), we participated in the BioNLP 2013 shared tasks, addressing the GENIA (GE) and the Cancer Genetics (CG) event extraction tasks. While the GE task focused on 13 molecular biology related event types concerning the protein NF-*κ*B, the CG task targeted a challenging group of 40 cancer biology related event types and involved 18 kinds of biological entities describing the development and progression of cancer. This poses an additional challenge to event extraction systems as they should be able to associate molecular level entities and events with anatomy level effects and organism level outcomes of cancer biology.

We recently proposed a novel system [4] for identifying relations and events concerning genes or gene products in the biomedical literature. The extraction method is based on the search for an approximate subgraph matching (ASM) between key context dependencies of events and graphs of input sentences. The performance is in line with the top systems in the GE task of the BioNLP- ST 2011 when evaluated on the 9 types of biological events. In the BioNLP- ST 2013, in addition to generalizing our system to investigate the two tasks, we attempted to integrate semantics into the graph matching scheme of the system using a distributional similarity model for more events. Considering that the all-paths graph representation adopted by Support Vector Machines (SVM) has led to the state-of-the-art performance in extracting drug-drug [5] and protein-protein interactions [6], we also evaluated the event extraction impact of using paths of all possible lengths among event participants as key context dependencies beyond using only the shortest paths in our system. We achieved a 46.38% F-score in the CG task, ranking 3*^rd ^*and a 48.93% F-score in the GE task, ranking 4*^th ^*[7].

After the 2013 challenge, we further explored three other ways to extend our ASM-based system in our previously published work. First, we allow non-essential nodes to be skipped, and incorporated a node skipping penalty into the subgraph distance function of the ASM algorithm. The previous design allows variations in edge attributes such as labels and directionalities but requires each node in a pattern graph to find its injective match in a sentence graph. While this requirement preserves the complete lexical context of an annotated event in the pattern, it retains terms specific to a particular event expression but non-essential to the underlying meaning of the event, thus affecting the generalizability of the pattern. For instance, "activity" in a cascaded *Positive_regulation *pattern "induction of binding activity" is redundant as well as "gene" in a *Regulation *pattern "regulated BIO_Entity gene". Protein/gene mentions are anonymized using "BIO_Entity" to ensure generalization. We therefore conjecture that allowing non-essential nodes in patterns to be skipped during graph matching can help to retrieve more events.

Second, we learned from training data an individual subgraph distance threshold for each event pattern. The previous design assigns a unified threshold to all patterns of each event type. Compared to the batch threshold, we hypothesize that a customized threshold can capture more precisely the variation tolerance of each pattern, and thus contribute to the event extraction precision. Third, we implemented the well-known empirical risk minimization (ERM) principle [8] to optimize the event pattern set. In contrast to the previous optimization module that measures each pattern in terms of its prediction precision, the new approach evaluates each pattern in terms of both wrong and missed event predictions and balances the prediction errors on training data against regularization. We hope that the ERM-based optimization approach can result in a pattern set that is more generalizable to unseen data.

We organized the rest of the paper as follows: In Section 2, we briefly introduce our graph matching based event extraction system. We describe in Section 3 our experiments during the 2013 challenge and attempted extensions after the challenge. Some implementation details are elaborated in Section 4 and our results and discussion are presented in Section 5. Finally, we summarize the paper and introduce future work in Section 6.

## ASM-based event extraction

The BioNLP shared task data include annotations for several different event types. The structure of each event is defined to include the type of the event, a "trigger" word that introduces the event, and the arguments of the event (such as the theme or the cause of the event). The arguments will typically be a biological entity introduced in the text, or another event that has been extracted.

We apply a machine learning approach based on the concept of instance-based reasoning [8] that takes advantage of consistencies in the linguistic expression of events, and specifically considers the syntactic dependencies that exist among the components of the events, namely the triggers and the arguments. The objective is to learn patterns of syntactic dependencies that connect these components. These patterns can be matched to the dependency graphs of new input sentences to identify relevant events in those sentences. This identification is achieved through the application of a matching algorithm called approximate subgraph matching (ASM) that we developed previously [4]. The method incorporates a tolerance for error in the process of matching patterns to graphs, and as a result is able to retrieve events in varying syntactic contexts while maintaining a high level of precision. The approach has been evaluated on a range of event and relation extraction tasks, and has achieved performance competitive with other systems. More details are available in the original publication [4]; the ASM algorithm itself has been released open source at http://asmalgorithm.sourceforge.net/.

**Figure 1:**
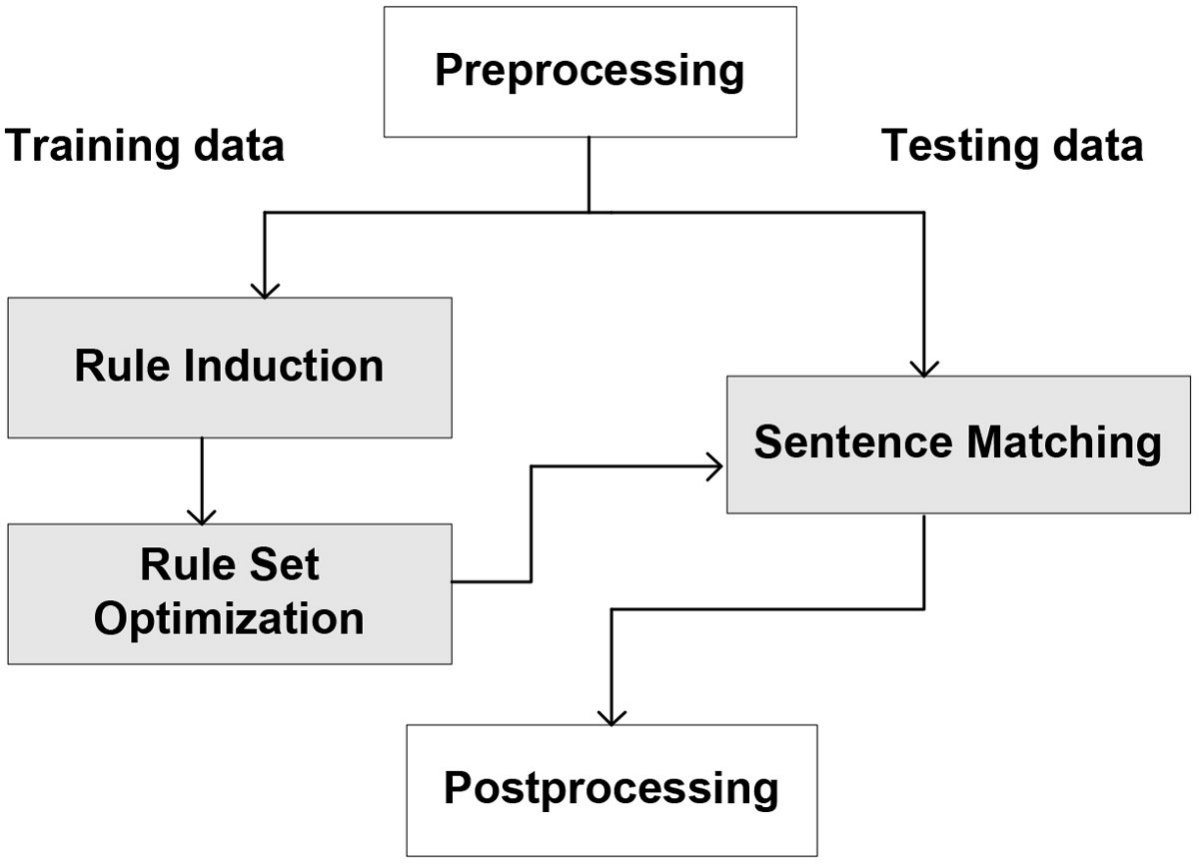
**ASM-based Event Extraction Framework**.

The architecture of the ASM-based system appears in Figure 1. There are three primary components, (1) pattern induction, (2) matching of patterns to sentences, and (3) optimization of patterns for the event extraction task. The method targets events that are expressed in the scope of a single sentence and assumes that the core entities that play a role in the event (e.g., proteins or genes) have been annotated in a preprocessing step. Below, we describe the components in more detail.

### Pattern induction

The objective of this step is to learn patterns corresponding to provided events annotated in training data, represented as a subgraph in a dependency parse of a sentence. This step takes as input a dependency graph representing a training sentence, and identifies the shortest path in that graph that connects the annotated trigger word to each event argument. We focus strictly on the shortest path under the assumption that it contains the strongest information about the connection between the components of the event [9-11]. This focus on the syntactic relationships, and the lexical items in the path connecting the components, is in contrast to other approaches to event extraction that make use of a broader range of linguistic evidence in the sentence, ranging from individual words or sequences of words (n-grams), to the presence of semantic concepts that have been pre-identified [6,12,13].

To facilitate generalization of the syntactic patterns to new sentences involving different specific entities, annotated named entities are replaced with a generic string representing the type of the entity (e.g., "Protein", "Organism" or "Cellular component"). This ensures that the particular lexical items filling the event argument roles are abstracted out of the induced patterns.

Alongside the graph representation of each event, the correspondences of the elements of the graph to the target event representation are recorded. This indicates the specific event type that the graph corresponds to, as well as which nodes in the graph correspond to specific event arguments, and their semantic role with respect to the event. This is utilized after pattern matching to produce an event representation for a new sentence from the matched pattern.

Directionality of the graph is ignored in the pattern induction process. In the case that there are multiple paths of the same (shortest) length in the dependency graph, we consider all of them. Where there are trigger words that consist of multiple lexical tokens, we extract only paths that connect all of the tokens simultaneously. For complex events where an event argument is itself an event, the shortest path that connects the trigger word of the main event to the trigger word of the sub-event is utilized.

For a complex event that has multiple arguments, we take the union of all of the shortest dependency paths from a trigger to each event argument, identifying a graph consisting of all event participants. We additionally preserve the individual dependency paths to enable separate extraction of specific event arguments. Where the arguments share a common event trigger word, they are grouped together. These two approaches are complementary: the use of individual paths aims to increase recall of potential events, while the path unions increase precision through joint inference.

**Figure 2:**
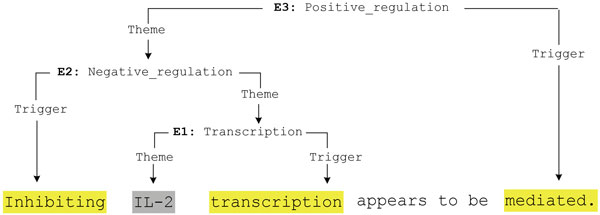
**Event Pattern Induction Example**.

Figure 2 provides an example of the pattern induction process, starting with a sentence annotated with a *Positive_regulation *event, derived from the publication PMC-1134658. Annotations for the basic entities (proteins) and the event trigger are included in the figure. A dependency graph for the sentence is produced using the McClosky-Charniak domain-adapted parser [14], and paths that connect the event triggers are identified. There are two paths connecting the tokens "lead-20/VBP" and "ligation-6/NN", so both are considered. Five distinct event patterns are inferred from this single example, listed in Table 1. The graphs captured in "E1a" and "E1b" are unions of paths, and therefore subsume the individual paths captured in the other patterns.

**Table 1:** Event pattern representation.

Pattern ID	Pattern Description				Graph Representation
	**Type**	**Trigger**	**Theme**	**Cause**	

E1a	Pos. reg.	lead-20/VBP	Phosphorylation: phosphorylation-23/NN	Binding: ligation-6/NN	nsubj(lead-20/VBP, ligation-6/NN) prep_to(lead-20/VBP, phosphorylation-23/NN)

E1b	Pos. reg.	lead-20/VBP	Phosphorylation: phosphorylation-23/NN	Binding: ligation-6/NN	rcmod(ligation-6/NN, lead-20/VBP) prep_to(lead-20/VBP, phosphorylation-23/NN)

E1c	Pos. reg.	lead-20/VBP	Phosphorylation: phosphorylation-23/NN		prep_to(lead-20/VBP, phosphorylation-23/NN)

E1d	Pos. reg.	lead-20/VBP		Binding: ligation-6/NN	nsubj(lead-20/VBP, ligation-6/NN)

E1e	Pos. reg.	lead-20/VBP		Binding: ligation-6/NN	rcmod(ligation-6/NN, lead-20/VBP)

### Sentence matching

To identify an event in a test sentence, the sentence is parsed into its dependency representation, and matched to the patterns learned from the training data. Once a match has been identified, the event representation is populated from the relevant correspondences between the pattern graph and the event semantics associated to the pattern. In this approach, event recognition reduces to a (dependency) subgraph matching problem. We proposed a novel *approximate subgraph matching *(ASM) algorithm, which identifies a subgraph in a test sentence graph isomorphic to a pattern graph, to perform this matching in prior work [4]. This algorithm is defined as follows.

**Definition 1**. An event pattern graph *G_r _*= (*V_r _, E_r_*) is *approximately isomorphic *to a subgraph *S_s _*of a sentence graph *G_s _*= (*V_s_, E_s_*), denoted by *G_r _*≅ *S_s _⊆ G_s_*, if there is an injective mapping *f *: *Vr → Vs *such that, for a given threshold *t, t ≥ *0, the subgraph distance between *G_r _*and *G_s _*satisfies 0 *≤ *subgraphDist*f *(*G_r_, G_s_*) *≤ t*, where subgraphDist*f *(*G_r_, G_s_*) = *w_s _× *structDist*_f _*(*G_r_, G_s_*)+*w_l _× *labelDist*_f _*(*G_r_, G_s_*)+ *w_d _× *directionalityDist*_f _*(*G_r_, G_s_*).

To allow for some variations to exist between the sentence graph and the pattern graph, we introduce three measures that each captures one kind of variation between the graphs. The measure **structDist **accumulates structural differences (formalized as a difference in the path length), **labelDist **counts differences in the edge labels, and **directionalityDist **tracks differences in the edge directionality. Each measure is computed for the path connecting a pair of nodes in the pattern graph, compared with the corresponding pair of nodes in the sentence graph, where the corresponding nodes are determined by the alignment of the graphs that results in the minimal structural difference with the pattern graph. Each of these measures is given a non-negative weighting in the algorithm (*w_s_, w_l _*and *w_d_*, respectively).

By default, these weights are set to be equal; however, they can be tuned to emphasize some differences over the others. A distance threshold *t *controls the amount of divergence between two graphs that is allowed, and controls isomorphism quality. Smaller values of *t *result in stricter, more closely isomorphic matching. Larger values of *t *allow for more variation, enable matching in more complicated sentences and generally lead to increased recall of events from the test sentences. However, it also requires evaluation of more possible matches and can therefore incur a larger search cost.

**Figure 3:**
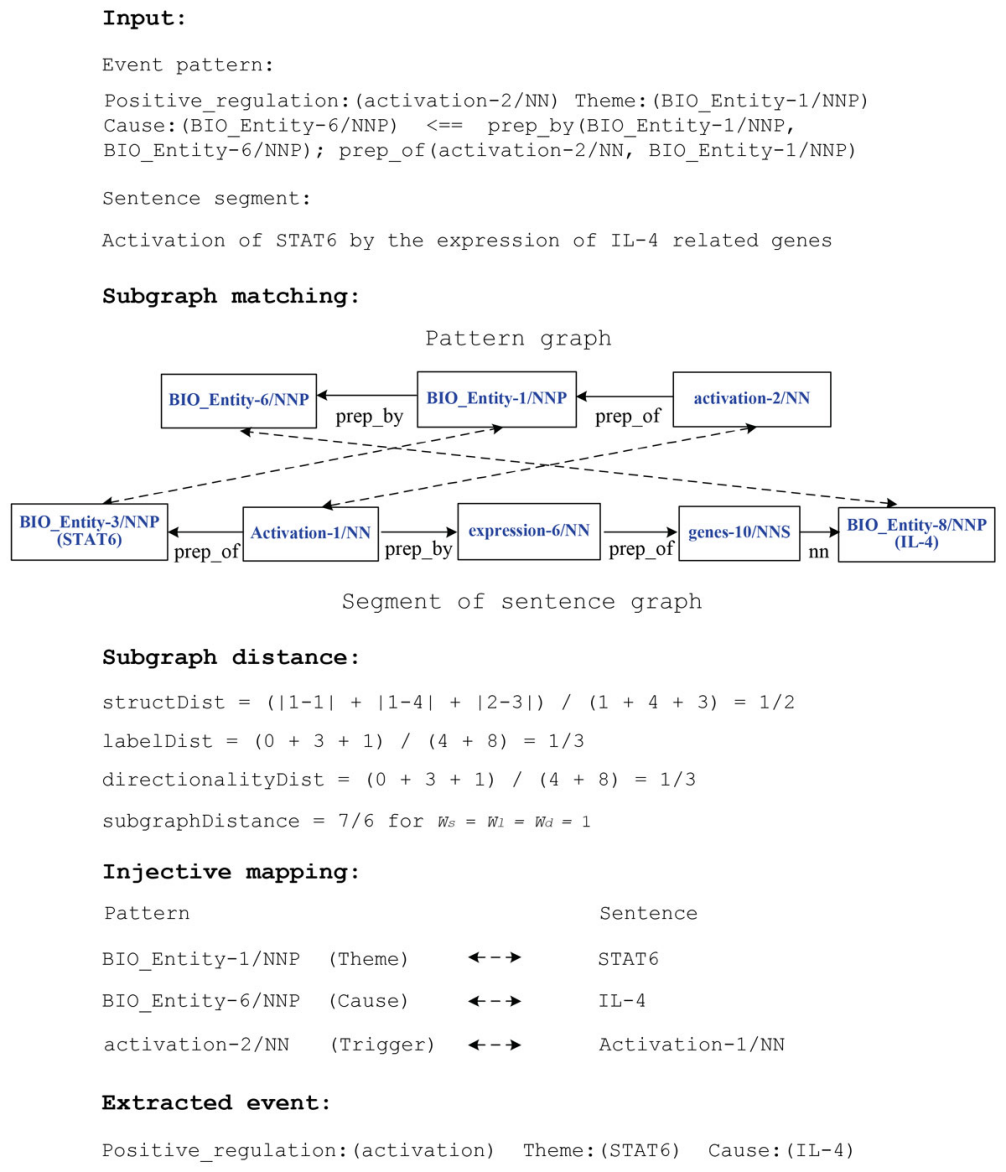
**ASM-based Event Extraction**.

An example of event extraction using ASM is presented in Figure 3. Again, we use the McClosky-Charniak parser [14] to parse the sentence, and attempt to match the sentence graph to the pattern graph for the *Positive regulation *event. In order to support a match between a pattern node and a sentence node, their relaxed POS tags (P*, allowing a plural noun form to match with a singular, or various conjugated forms of a verb to match) and the lemmatized form (L, derived from application of the BioLemmatizer [15]) of the associated tokens must be identical ("P*+L" matching criteria).

**Figure 4:**
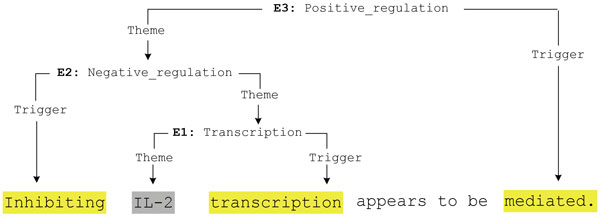
**Iterative Bottom-up Event Extraction Example**.

Pattern matching proceeds iteratively and bottom-up, to enable the extraction of complex and nested events. As illustrated in Figure 4, containing three chained events from a sentence (PMID-10229815), events which only take entities as arguments are matched first, and any matched events are available as potential arguments of higher-order events in subsequent pattern matching. The process ends when no further event candidates are produced for a test sentence.

Based on an intuition that the syntactic contexts that relate particular types of sub-events to a higher-order event are likely to generalize across event types, we do not constrain the type of a sub-event during higher-order event pattern matching. This increases the chance of the system extracting complex events with nested structures, while still respecting the syntactic constraints.

### Pattern set optimization

Like other instance-based reasoning systems, it is critical that the patterns we match to test sentences have high precision (avoiding false positive matches). For instance, the dependency between "TNF" and "mRNA" in a *Transcription *pattern derived from a sentence such as "expression of TNF mRNA" should not result in the extraction of a *Transcription *event for the phrase "level of TNF mRNA", even though they share a matchable dependency. We therefore implemented a strategy to evaluate the precision of each induced pattern *r_i_*, based on Equation (1) applied to test data. Patterns are ranked by *Prec*(*r_i_*); any patterns falling below an empirically determined threshold are removed from the pattern set.

(1) $$Prec\left(r_{i}\right)=\frac{\#correct\text{\_}predictions\text{\_}by\text{\_}r_{i}}{\#total\text{\_}predictions\text{\_}by\text{\_}r_{i}}$$

Due to potential down-stream effects of this filtering on nested patterns resulting from our iterative pattern matching process, we also applied the pattern set optimization process iteratively. In each iteration, an end-to-end evaluation process of matching over test sentences, pattern ranking and pattern filtering is performed. This results in an optimized pattern set that improves the overall precision of the event extraction enabled by our method.

## Extensions to event extraction system

We extended our previously proposed event extraction system [4] in several ways for the 2013 BioNLP shared task. First, we experimented with incorporating a distributional similarity model into the graph matching scheme to allow for more variation during matching, and second, we explored the use of dependency paths of all possible lengths (rather than only shortest paths) in the pattern induction phase.

We then explored additional changes to the approach in work subsequent to the 2013 shared task: (1) incorporate a node skipping penalty into the subgraph distance function of our approximate subgraph matching algorithm. (2) learn a customized threshold for each pattern. (3) implement the well-known empirical risk minimization (ERM) principle to optimize the event pattern set. Next, we elaborate these system experiments in detail.

### Experiments in BioNLP-ST 2013

#### Integrating distributional similarity model

As described above, the ASM algorithm employs a distance measure based on 3 dimensions of variance that can exist between two graphs. This allows for some differences to exist between two matched graphs. However, the node mapping that is performed between the graphs is based on strict lexical matching. In our previous work, we considered various criteria for node matching, including relaxing of the strict matching to consider token lemmas (L) or POS tags (P), or combinations such as "P*+L" introduced above. However, this still requires fairly tight alignment between a pattern graph and a sentence graph. We experimented with dropping any lemma matching requirement, and only using POS information, but observed a sharp drop in precision. Despite a nearly 14% increase in recall, the overall impact on F-scores was strongly negative [16]. This suggests that word-level information is an important component of matching in the framework of our system.

To allow for additional flexibility in word choice, we decided to explore a refinement of the node mapping strategies that takes lexical variation into consideration. This can be considered another dimension of variance to be supported in the algorithm, and would for instance allow a pattern token "crucial" to match a sentence token such as "critical" which could result in extraction of a relevant event. We previously attempted to allow for such lexical variation by allowing words to match their synonyms (as defined by WordNet [17]) [18]. However, since WordNet is developed for the general English language, it relates biomedical terms e.g., "expression" with general words such as "aspect" and "face", thus leading to incorrect events.

We therefore decided to experiment with a different approach to accommodating lexical variation during node matching, specifically by integrating an empirically-derived similarity model. We implemented a distributional similarity model (DSM); this model is based on the distributional hypothesis [19] that words that occur in the same contexts tend to share similar meanings. We expected that incorporating such a model would increase recall without impacting precision too much.

There have been many approaches to computing distributional similarity of words in a corpus [20,21]. The output is typically a ranked list of similar words to each word. We reimplemented the model proposed by [21], in which each word is represented by a feature vector and each feature corresponds to a context where the word appears. The value of the feature is the pointwise mutual information [22] between the feature and the word.

Let *c *be a context and *F_c_*(*w*) be the frequency count of a word *w *occurring in context *c*. The pointwise mutual information, *mi_w,c _*between *c *and *w *is defined as:

$$mi_{w,c}=\frac{F_{c}\left(w\right)}{\frac{\underset{i}{\sum }F_{i}\left(w\right)}{N}\times \frac{\underset{i}{\sum }F_{c}\left(j\right)}{N}}$$

where $N=\underset{i}{\sum }\underset{j}{\sum }F_{i}\left(j\right)$ is the total frequency count of all words and their contexts.

Since mutual information tends to be biased towards infrequent words/features, we multiplied the above mutual information value by a discounting factor as suggested in [21]. We then computed the similarity between two words via the cosine coefficient [23] of their mutual information vectors.

We tried two different strategies to integrate distributional similarity into our event extraction system. In the first strategy, DSM is applied at the node matching step, allowing a match between two unequal lexical items if the sentence token appears in the list of the top *M *most similar words to the pattern token. The second approach is generative and applies to event patterns. A copy of an event pattern is produced by substituting a pattern token with a similar term; this copying is performed for each of the top *M *most similar words. The first method results in a more general flexibility during event extraction, while the second method gives the opportunity to measure the impact of each possible token substitution in a pattern separately, and to filter out spurious synonyms during the pattern optimization step.

#### Adopting all-paths for event patterns

The ASM algorithm was designed to work with only the shortest path between event components [4]. However, there is a body of work that has explored the value of considering all paths in a dependency graph for tasks such as extraction of protein- protein interactions (PPI) [6], event extraction [12], and drug-drug interactions [24]. The latter system, using an all-paths graph kernel, won the recent DDIExtraction 2011 challenge [25]. The kernel includes two representations for each sentence with a pair of interacting entities, the full dependency parse and the linear token sequence. At the expense of computational complexity, this representation enables the kernel to explore the full dependency graph, and thereby the broader sentential context of an interaction.

The shortest dependency path may not provide sufficient syntactic context to enable precise relation extraction. Therefore, borrowing from the all-path graph representation, we experimented with extending the representation used by the ASM algorithm in the pattern induction step to consider acyclic paths of all possible lengths among event components.

### Experiments after BioNLP-ST 2013

#### Incorporating node skipping penalty into ASM

As shown in **Definition 1**, the subgraph distance design in our system [4] considers variations in edge labels and edge directionalities but insists that a candidate match should possess an injective mapping between nodes of a pattern graph and a sentence graph.

Preserving the complete lexical contexts of an annotated event in the induced pattern has the advantage of achieving precise predictions. However, it often retains terms from a particular textual expression of an event but in fact not essential to the underlying meaning of the event. For instance, the dependency context "induction of binding activity" of a pattern encodes the context of a *Positive_regulation *event cascaded with a lower order *Binding *event. Since the term "binding" indicates a binding activity by itself, the additional "activity" is redundant. Similarly, the term "gene" in the dependency context of a *Regulation *event pattern "regulated BIO Entity gene" is neglectable when the "BIO Entity" itself has been pre-annotated as a gene. Therefore, we hypothesize that providing an option in graph matching to skip the non-essential context words encoded in patterns can improve their generalizability.

We revised the subgraph distance function proposed in [4] by adding in a **nodeDist **measure which penalizes the number of skipped non-essential nodes normalized by the total number of pattern graph nodes for each candidate match between pattern and sentence graphs. In our experiments, essential context nodes of a pattern are considered to be the nodes corresponding to event triggers and event arguments such as theme or cause. The sub-event trigger is also considered for patterns that encode cascaded events.

Consequently, the original injective mapping *f *: *Vr → Vs *as in **Definition 1 **is relaxed to be $f\prime :\to V_{r}^{\prime }\to V_{s}$ where $V_{r}^{\prime }$ is a set of essential context nodes in a pattern graph. A candidate match can be considered only if a *f*'' exists between two graphs. In case that the original node injective mapping constraint is satisfied, i.e., no pattern node is skipped, **nodeDist **becomes 0 and the new distance function is equivalent to the original function. Similar to the weights *w_s_, w_l _*and *w_d_*, the non-negative weight *w_n _*can be tuned to accommodate the emphasis on **nodeDist **in the distance function. The new function is defined as follows.

subgraph $Dist_{f}^{\prime }$ (*G, Gs*) = *w_s _× *structDist*_f _*(*G_r_, G_s_*) + *w_l _× *labelDist*_f _*(*G_r_, G_s_*) + *w_d _× *directionalityDist*_f _*(*G_r_, G_s_*) + *w_n _× ***nodeDist***_f _*(*G_r_, Gs*), where

$$\text{modeDis}{\text{t}}_{f}\left(G_{r},\;G_{S}\right)=\frac{\#\left\{V_{r}-V_{r}^{\prime }\right\}}{\#V_{r}}.$$

#### Learning individual distance threshold for each event pattern

In the original design of our system [4], a unified subgraph distance threshold is assigned to all patterns of the same event type. Since the encoded graphs are different across the patterns, it is difficult for an event type-wise, batch threshold to precisely capture the graph variation tolerance of each pattern. Thus, we conjecture that an individual threshold would be more appropriate to regulate the subgraph retrieval quality of each pattern, thus improving the event extraction precision.

For patterns encoding lower order events, i.e., events that do not contain nested sub-events, learning a customized threshold is straightforward because their prediction results can be individually assessed. For a given threshold range, we can iteratively search for a threshold leading to the maximum performance of a pattern. The threshold is updated only if the current value results in a larger number of correct event predictions and an equivalent or better prediction precision. To alleviate the potential overfitting problem, a held-out data set is used to validate the candidate threshold before finalizing each update.

The same approach, however, cannot be applied to patterns encoding higher level events as individually measuring their performance is not feasible. Patterns nested with lower order sub-events depend on the corresponding lower order patterns, while patterns cascaded with higher order sub-events rely on all the patterns involved in the downstream, nested structures. Instead of tracing the hierarchical event correlations to evaluate each higher order pattern, we adopted a holistic approach to learn individual thresholds using a genetic algorithm (GA) [26] that automatically determines the values for higher order patterns by evaluating the entire event pattern set.

Our GA works with a population of potential threshold settings. Given a threshold range, the GA simultaneously assigns a candidate threshold value to each higher order pattern. The fitness function of GA evaluates the performance of the whole pattern set under the current threshold settings. The individually learned thresholds of lower order patterns remains untouched in the GA and the events produced by them serve as potential arguments to contribute to the functioning of higher level patterns. The GA iterates the fitness function and eventually returns a threshold setting that maximizes the F-score on the training data. Algorithm 1 formalizes our approach for learning the individual distance threshold for event patterns.

When evaluating pattern performance under different threshold settings, graph matching between patterns and sentences is performed only once with an assignment of the maximum candidate threshold to all patterns. By maintaining information on event predictions and corresponding pattern thresholds together, performance of various threshold settings can be efficiently computed. This is important for the GA especially when a large number of generations or population size is specified.

**Algorithm 1 **Pattern Threshold Learning Algorithm

**Input: **Dependency graphs of training and held-out sentences *Gt *and *Gh*; A finite set of event patterns *P *= {*p*_1_*, p*_2_*, · · ·, p_i_, · · ·*}, composed of lower order pattern subset *P_i _*and higher order subset *P_h_*; A predefined threshold value search range *V *= (*v_min_, v_i_, · · ·, v_max_*).

**Output: **A finite set of thresholds for patterns *T *= {*t*_1_*, t*_2_, · · · *, t_i_, · · ·*}.

1: **for all ***p_i _∈ P_l _***do**

2:     **for all ***v_i _∈ V ***do**

3:         **if **updateSinglePattern(*p_i_, v_i_, G_t_, G_h_*) is satisfied **then**

4:             *t*_i_*← v*_i_

5:         //updateSinglePattern() evaluates the individual performance of *p_i _*with threshold *v_i_*, and

6:         //*t_i _*updated only if *v_i _*results in more correct predictions and an equivalent or better precision

7: *T_h_ ← *geneticAlgorithm(*P, T_l_, G_t_, V *)

8: //geneticAlgorithm() undergoes procedures of selection, crossover and mutation, and returns an optimized threshold setting *T *for *P *by evaluating *P *as a whole

9: **return ***T*

#### Pattern set optimization by empirical risk minimization algorithm

The original pattern set optimization module [4] measures the prediction precision of patterns, and iteratively eliminates patterns whose precision is lower than an empirical threshold. We consider that the optimal event pattern set should satisfy the following three criteria: (1) maximum number of matches; (2) fewest number of prediction errors; and (3) least redundancy in patterns. Obviously, these criteria cannot be met simultaneously. Considering that the total number of prediction matches by the pattern set has been decided when the individual threshold of each pattern is learned, our optimization task becomes one of finding the best balance between the criteria (2) and (3).

We implemented the well-known empirical risk minimization (ERM) principle [8,27] to optimize the event pattern set by balancing prediction errors on training data against regularization on the overall redundancy of the pattern set. The objective function of our problem is shown in in Eq.(3).

(3) $$f\left(P\right)=E\left(P,\;G\right)+\lambda C_{p}\geq 0$$

*E*(*P, G*) in Eq.(4) models the prediction errors including both wrongly predicted and missed events, produced by a pattern set *P *evaluated against the gold annotation *G*.

(4) $$E\left(P,\;G\right)=N_{wrong}+N_{missed}$$

*C_p _*accumulates the information redundancy of each *p_i _∈ P *, measured by the percentage of non-essential nodes $\frac{\#\left\{V_{r}-V_{r}^{\prime }\right\}}{\#V_{r}}$ in *p_i_*, and *λ *is a regularization parameter that determines the degree of the penalty on the total redundancy.

Therefore, given an input pattern set *P*, our optimization problem is to find a pattern set P^*^ ⊂ P, which satisfies $P*\subset \text{arg}\underset{P\prime \subset P}{\text{min}}f\left(P\prime \right)$, where *P*' is a subset of *P*. Clearly, minimizing *f *(*P *) prefers compact and effective patterns encoding event arguments in an adjacent context, and penalizes the redundant information in complex patterns.

For our problem, a greedy backward elimination feature selection method is implemented, in which each pattern is evaluated according to its impact on the entire pattern set *P*, and the one whose removal incurs the largest reduction in *f *(*P*) is removed in each iteration. The optimization terminates when *f *(*P*) cannot be further reduced. Algorithm 2 shows the detailed procedure.

With *λC_p_*regularizing the optimization, the final set P^*^may not be the best pattern set in terms of minimizing the prediction errors on training data, but has better generalizability on unseen data.

**Algorithm 2 **ERM-based Pattern Set Optimization Algorithm

**Input: **A finite set of event patterns *P *= {*p*_1_*, p*_2_*, · · ·, p_i_, · · ·*}, where the distance threshold *t_i _*of *p_i _*is fixed.

**Output: **An optimized pattern set P^*^.

1: *P_c_ ← P *// *P_c_* is the current pattern set

2: **while ***P_c_* is not empty **do**

3:     compute *f *(*P_c_*)

4:     *maxGain *= 0

5:     **for all ***p_i _∈ P_c _***do**

6:         *P_t_ ← P_c_ − *{*p_i_*}

7:         Δ*f *= *f *(*P_c_*) *− f *(*P_t_*)

8:         **if **Δ*f > maxGain ***then**

9:             *maxGain *= Δ*f*

10:             p^*^ ← p_i_

11:     **if ***maxGain ≤ *0 **then**

12:         go to Line 14

13:     *P_c _← P_c _− *{p^*^}

14: P^*^ ← P_c_

15: **return **P^*^

## Implementation

### Experiments in BioNLP-ST 2013

#### Data preprocessing

The BioC project [28] provides a unified BioC XML format to address the interoperability issue among existing text mining tools. The shared task organizers provided the preprocessed data in the BioC [28] compliant XML format as supporting resources. We used the provided text analyses such as tokenization, sentence segmentation, POS tagging and lemmatization. For the syntactic analysis, considering that different syntactic parsers use different underlying approaches to analyze text, we employed both the Stanford parser [29] and the McClosky-Charniak-Johnson (Charniak) parser [14] to take advantage of the structural analysis of sentences from multiple possible views. The Stanford parser performs joint inference over the results of a lexicalized dependency parser and an unlexicalized Probabilistic Context-Free Grammar (PCFG) parser. The Charniak parser conducts *N*-best parse reranking over a lexicalized PCFG model. According to a recent evaluation [30] on parsers that are trained using the GENIA Treebank corpus, both parsers achieve the state-of-the-art performance on the biomedical text. In our experiments, training sentences are parsed by both parsers to produce dependency graphs for event pattern induction while test sentences are parsed by the Charniak parser only for event extraction.

#### ASM parameter setting

For the GE task, the ASM requires 16 parameters. In addition to the distance function weights *w_s_, w_l _*and *w_d_*, an individual threshold *t_e _*is assigned to each of the 13 event types as they are likely to possess different event contexts. Likewise, the ASM requires 43 parameters for the CG task. We inherited the previous ASM parameters [4] determined on the 2011 GE task training data using a genetic algorithm (GA) [26], and adapted them to the 2013 tasks in terms of the event type and the configuration of argument. For example, the same *t_e _*of the "Binding" events in the GE task is assigned to the "Pathway" events in the CG task as the two event types share similar argument configurations.

The parameter setting of the 2013 GE task is presented in Table 2 with the equal weight constraint *w_s _*= *w_l _*= *w_d_*. The graph node matching criterion "P*+L" requires the relaxed POS tags and the token lemmas to be identical. We observed that it demonstrated a superior performance among all the matching criteria, and thus we used it in the ASM.

**Table 2:** ASM parameter setting in the 2013 GE task.

Parameter	Value	Parameter	Value
*tGene expression*	8	*tUbiquitination*	3
*tTranscription*	7	*tBinding*	7
*tProtein catabolism*	10	*tRegulation*	3
*tPhosphorylation*	8	*tPositive regulation*	3
*tLocalization*	8	*tNegative regulation*	3
*tAcetylation*	3	*ws*	10
*tDeacetylation*	3	*wl*	10
*tProtein modification*	3	*wd*	10

#### Distributional similarity model

Based on Pantel's distributional similarity model [21], we had the following modifications in our implementation: (1) instead of surface words, we used lemmas generated by the BioLemmatizer [15] along with their POS information for better generalization and category disambiguation. (2) we took advantage of dependency contexts where words appear rather than their linear contexts. Besides dependent tokens, the dependency type and the directionality are also captured from dependency graphs. For example, "toxicity*→*amod" is a feature of the token "nonhematopoietic JJ". While we only included the first-level dependencies of a word in the model, contexts of multiple dependency depths can be flexibly used in our implementation. (3) we scaled the resulting *mi_w,c_* into the 0[1] range using $\frac{\lambda ,mi_{w,c}}{1+\lambda \cdot mi_{w,c}}$ as unnormalized, greater $mi_{w,c}$ values may dominate the similarity computation between words. Empirically, a value of *λ *= 0.01 is used.

Compared to exisiting biomedical corpora that focus on particular biological domains or topics, PubMed abstracts cover a much wider range of words and capture their diverse usage contexts in biomedical texts. Therefore, we randomly selected 5 million abstracts from the whole PubMed and built our distributional similarity model based on the random selection. To concentrate on representative context vectors for *w*, in the computation we only consider *mi_w,c_* for which *c *appears more than 5 times. The final model is composed of 2.8 million distinct tokens and 0.4 million features. When an amino acid such as "lysine" is queried to both the original Pantel model and our modified model, the top 15 tokens in the ranked list produced by our model are all correct amino acid names.

### Experiments after BioNLP-ST 2013

#### Pattern threshold learning

Two empirical search ranges of integer values: [0,12] and [0,6] are used in Algorithm 1 for learning the individual threshold of lower order patterns and higher order patterns, respectively. A threshold higher than 12 for lower order patterns may produce more correct predictions at the expense of precision. However, the incurred lower level false positive events will be propagated to the subsequent, recursive prediction of higher level events, leading to a detrimental error accumulation.

Determining thresholds of lower level patterns through explicit, individual search allows the GA to focus on the combinatorial effects of thresholds of higher order patterns from a holistic perspective. We set up the GA to evolve for 100 generations.

Each generation consists of a population of 100 combinations of potential thresholds. Starting with a random population of 100 potential solutions, GA proceeds until it reaches 100 generations. The population size and the number of generations are decided with consideration of the runtime cost of evaluating the fitness function. A large population size or a large number of generations would incur an expensive runtime cost of evaluation. An equal weights *w_s _*= *w_l _*= *w_d _*= *w_n _*constraint is used throughout our experiments performed after BioNLP-ST 2013.

#### Pattern set optimization

The regularization parameter *λ *is determined by optimizing *λ *on the training dataset and testing on the development dataset. Using the best value of *λ *in the previous step, we obtained the final event pattern set on the union of the training and development sets.

According to Algorithm 2, it is time-consuming to re-evaluate the entire pattern set *P_c_* in each iteration. In our implementation, we actively traced the patterns potentially impacted by the removal of p^*^, so the algorithm is efficient by re-evaluating in each iteration only a small subset of *P_c_*.

## Results and discussion

In this section, we respectively report our performance on the GE task and the CG task. We focus on the GE task to discuss the performance of the event extraction system and our attempted extensions both in and after the 2013 shared task.

### GE task

#### Datasets

The dataset of the 2013 GE task is composed of full-text articles from PubMed Central. In terms of sections, the task organizers divided articles into smaller segments [31]. The statistics of the GE dataset is shown in Table 3.

**Table 3:** Statistics of BioNLP-ST 2013 GE dataset.

Attributes Counted	Training	Development	Test
Full article segments	222	249	305
Proteins	3,571	4,138	4,359
Annotated events	2,817	3,199	3,301(hidden)

To be consistent with the 3:1 training/development data ratio in previous GE tasks [1,2], we combined the development and the training sets, and reshuffled the data randomly to create a training/development division of 353/118. We report hereafter results on the training/development data using the new partition.

#### GE results on development set in BioNLP-ST 2013

Table 4 presents the results on the development data using event patterns from different parsers. We have removed patterns that possess isomorphic graph representations detected by an Exact Subgraph Matching (ESM) algorithm [16], and reported only the numbers of optimized, unique patterns. The ensemble pattern set that contains patterns from both parsers obtains a superior result over using an individual parser. It is understandable that the Charniak parser produces a performance close to the ensemble performance as event extraction is performed on sentences parsed by the Charniak parser. We used the ensemble pattern set in the experiments.

**Table 4:** Performance using different parsers on the development set.

Parser Type	Event pattern	Recall	Precision	F-score
Charniak	2,923	47.01%	66.01%	54.91%
Stanford	3,305	43.66%	67.67%	53.08%
Ensemble	4,617	47.45%	65.65%	55.09%

When the distributional similarity model (DSM) is used in graph matching, except biological entities, we granted a node match as long as a pattern token is in the list of top *M *most similar words of a sentence token. "DSM 3" represents the top 3 similar words according to the DSM. Further, for comparison we applied DSM to trigger tokens only, as shown in Table 5.

**Table 5:** Performance of integrated DSM on development set.

All Tokens	Recall	Precision	F-score
DSM 1	47.98%	52.56%	50.17%
DSM 3	48.68%	35.07%	40.77%
DSM 10	53.43%	19.38%	28.44%

**Trigger Tokens**	**Recall**	**Precision**	**F-score**

DSM 1	48.06%	54.22%	50.95%
DSM 3	48.59%	37.00%	42.01%
DSM 10	53.35%	24.65%	33.72%

We observed that with the DSM the recall is significantly improved to 53.43%. However, a substantial precision decrease results in an unfavorable F-score lower than the ensemble baseline in Table 4. After looking into the specific graph matches, we realized that a large number of the false positive events come from antonyms generated by the DSM because they always appear in same contexts. As a result, for instance DSM produces "decrease" and "low" as the most similar words for "increase" and "high". Automatically removing the antonyms deserves further investigation in our future work. When the top *M *most similar words are used to generate additional patterns, while the optimization process ensures the extraction precision, the recall does not increase as we expected. The introduced false positive regulation-related events offset the recall gain from non-regulatory events. As a result, we obtained a performance comparable to the baseline.

Yih *et al*. [32] recently proposed a method named PILSA by introducing a polarity-inducing vector space representation into the traditional latent semantic analysis to automatically identify antonyms. With the help of a discriminative training, PILSA significantly outperforms the previous methods. While the method was proposed to handle antonyms in general English, considering that the antonym problem we encountered is mostly related to verbs and adjectives, we are interested in applying it to our biomedical context in the future. In addition, instead of choosing the top *M *most similar words, we will consider thresholding the selected DSM variants by DSM similarity in future work. This might alleviate the precision problem as some words do not necessarily have close synonyms while others have many.

As shown in Table 6 compared to the shortest paths, using all-paths does not lead to a substantial F-score increase in our event extraction system. However, due to that the number of patterns is more than doubled, the runtime cost of pattern optimization is significantly increased. In fact, the optimization process eventually discarded a large number of all-paths patterns. We consider that since the relation-signaling words have been annotated as triggers in the event extraction task, they are naturally included into our shortest path-based patterns. This contrasts with the motivation of the all-paths graph representation proposed for binary relation problems [6] in which relation-signaling words are often missed on the shortest paths unless broader contexts are explored. This partially explains why using all-paths did not lead to a significant increase.

**Table 6:** Performance of using all-paths on development set.

Path Type	Event Pattern	Recall	Precision	F-score
All-paths	9,527	48.77%	64.64%	55.59%
Shortest paths	4,617	47.45%	65.65%	55.09%

#### GE results on development set after BioNLP-ST 2013

The core components in our original [4] and the extended systems include, respectively: (1) ASM without node skipping; (1') ASM with node skipping; (2) event type-wise, batch pattern threshold; (2') individual pattern threshold; (3) precision-based optimization; and (3') ERM-based pattern set optimization. Therefore, the event extraction system used in BioNLP-ST 2013 can be represented by "1 + 2 + 3", and the extended system after the shared task denoted by "1' + 2' + 3'". Table 7 provides the results on the GE development dataset under various combinations of system components to demonstrate the impact of different settings. Parameters in Table 2 are used for the batch threshold setting. For compactness, we use the index to denote the corresponding system component hereafter.

**Table 7:** Performance comparison on development set under various settings.

System Setting	Event Pattern	Recall	Precision	F-score
1 + 2 + 3	4,617	47.45%	65.65%	55.09%
1*l *+ 2 + 3	4,593	49.21%	64.48%	55.82%
1*l *+ 2 + 3*l*	4,533	48.50%	67.36%	56.39%
1 + 2*l *+ 3*l*	4,787	45.60%	72.14%	55.88%
1*l *+ 2*l *+ 3*l*	4,806	46.83%	71.89%	56.72%

Compared to our performance reported in BioNLP-ST 2013, our new extensions together improve the overall F-score by 1.6%, with a 0.6% drop in recall but a significant 6% increase in precision. Careful examination on the prediction results confirms that the precision improvement primarily comes from extensions 2' and 3'.

The individual threshold is customized for each pattern and helps to reduce the prediction errors of each pattern by capturing more precisely the individual variation tolerance. As a result, more patterns are retained in the final pattern set, rather than being eliminated by the optimization process when using the type-wise, batch threshold. The ERM-based pattern set optimization also contributes to the noticeable 72% precision by modeling the prediction errors more effectively as compared to the precision-based optimization using a predefined threshold. We observed that patterns possessing a prediction precision above ¼, which would have been preserved in the original system [4], were actually removed by the ERM-based optimization after assessing their prediction errors and information redundancy. However, the individual threshold incurs a drop in recall. As observed in Table 7 settings that possess batch threshold generally achieve a higher recall than the ones using individual threshold.

We noticed that the node skipping extension to the original ASM algorithm mostly contributes to extracting higher order events rather than lower level events. Based on our observation, lower order events are generally described in a narrow context composed of a focused set of contextual words. For instance, without considering event triggers and participating biological entities, only 56 different contextual words are used across all *Transcription *event patterns from training data, such as "transcript", "amount", "marker" and "mRNA". When a *Transcription *event is mentioned in text, it is always characterized by these context words. As a result, skipping contextual nodes in these patterns harms their extraction precision because the meaning of the encoded event becomes erroneous, leading to the removal of the patterns during optimization. In this case, essential contextual nodes in patterns are beyond our definition that includes only event triggers and event arguments.

On the contrary, the description of higher order events involves a much broader context consisting of an open set of contextual words. For example, we extracted 300 non-essential nodes across all *Positive_regulation *event patterns, involving words like "vector", "role", "ability", "activity" and "level". This is understandable because higher order events in text describe various kinds of interactions among other events, and thus authors' choice of words tends to be diverse and flexible. Compared to the indispensable role of event triggers that bridges the underlying sub-events, the weaker supporting role of other nodes in higher order patterns enables the possibility of leaving them out in the graph matching.

Since 1' + 2 + 3' and 1' + 2' + 3' achieve a better F-score than others on the development set, we focused on both settings to experiment on the GE task test data.

#### GE results on test set in BioNLP-ST 2013

Considering that the DSM and all-paths extensions do not lead to significant improvements, we applied the original system settings to extract events from the test dataset. Further, we took advantage of existing annotated resources by adding the 2011 GE task data [33] and the EPI (Epigenetics and Post-translational Modifications) task data [34] as additional training instances for relevant event types of the 2013 GE task. In the end, we obtained 14,448 event patterns from our training data across all event types. For one document, our system takes less than one second to match it with all patterns and produce extraction results.

Our NCBI submission ranks 4*th *among 12 different participating teams of the GE task, achieving a F-score of 48.93% on the 305 test documents. The overall performance of top 8 systems is presented in Table 8. Also, it provides a detailed performance comparison across different event types. "SVT" represents simple events that involve a trigger and a theme only; "PTM" denotes protein modification related events that possess an optional cause argument; "BIND" indicates *Binding *events that take participants of varying numbers; "REG" stands for regulatory events with complex semantic roles.

**Table 8:** Performance comparison among top 8 systems in 2013 GE task.

System	SVT	PTM	BIND	REG		TOTAL	
	**F1(%)**	**F1(%)**	**F1(%)**	**F1(%)**	**Recall(%)**	**Precision(%)**	**F1(%)**

EVEX	76.59	65.37	42.88	38.41	45.44	58.03	50.97
TEES 2.1	76.82	66.49	43.32	38.05	46.17	56.32	50.74
BioSEM	76.11	74.37	49.76	35.8	42.47	62.83	50.68
**NCBI**	72.55	70.45	39.56	34.25	40.53	61.72	48.93
DlutNLP	74.42	69.36	42.43	32.92	40.81	57	47.56
HDS4NLP	79.07	73.17	37.32	21.64	37.11	51.19	43.03
NICTANLM	64.66	53.64	31.61	29.63	36.99	50.68	42.77
USheff	64.86	55.68	37.7	30.18	31.69	63.28	42.23

Our performance is close to the best-performing systems "EVEX" [35] and "TEES 2.1" [36]. While recall and precision are generally adjustable and inversely related, our system shows an overall good precision. This suggests that automatically learned and optimized event patterns not only have a stable generalization to unseen text but also can identify events precisely.

Further, as shown in Table 9 we investigated the impact of the additional training instances from 2011 tasks and the ensemble pattern set from different parsers. The 2011 data increase our F-score by 3%, and help us become the only team that detected "Ubiquitination" events from test data. We observed that compared to using patterns from the Charniak parser alone, the Stanford parser induced patterns do not bring in additional benefits on the test data.

**Table 9:** Impact of 2011 data and ensemble pattern set in 2013 GE task.

System Attribute	Recall	Precision	F-score
Ensemble 2013 + 2011 data	40.53%	61.72%	48.93%
Ensemble 2013 data	35.63%	63.91%	45.75%
Charniak 2013 data	35.29%	65.71%	45.92%

#### GE results on test set after BioNLP-ST 2013

Similarly, we further incorporated the 2011 shared task data into our system and tested settings 1' + 2 + 3' and 1' + 2' + 3' on the GE task test data. Table 10 presents the detailed comparison across event types between our results during and after BioNLP-ST 2013.

**Table 10:** Performance comparison on GE test set under different settings.

System Setting	SVT		PTM		BIND		REG		TOTAL		
	**R**	**P(%)**	**R**	**P(%)**	**R**	**P(%)**	**R**	**P(%)**	**Rec.(%)**	**Prec.(%)**	**F1(%)**

1 + 2 + 3	72.99	72.12	64.92	77.02	37.54	41.81	24.74	55.61	40.53	61.72	48.93
1' + 2 + 3'	74.07	72.85	64.92	68.50	40.54	39.24	25.26	49.45	41.41	57.80	48.25
1' + 2'+ 3'	68.55	81.57	59.69	77.03	31.23	49.76	26.18	53.69	39.32	64.74	48.93

A similar trend to the results on the development set is observed on the test set. Our new extensions help to improve the extraction precision by 3% to 65%. However, the 1% drop in recall coincidentally results in a F-score identical to our performance in the shared task (1 + 2 + 3).

We observed that the regularization parameter *λ *plays an important role in the ERM-based optimization process. Its impact is primarily on patterns that possess multiple non-essential nodes and produce a small number of events. It determines the inclusion or removal of these patterns in the pattern set optimization. We optimized *λ *on the 2011 shared task data and validated it on the 2013 data. Eventually, *λ *= 6 and *λ *= 3 are used to produce the results for 1' + 2' + 3' and 1' + 2 + 3' in Table 10.

We also evaluated the individual impact of the 2011 data and 2013 data on the test set using the setting 1' +2' +3' as presented in Table 11. The 2011 data achieves a comparable performance to the 2013 data, and the extensions help to improve the F-score by 0.8% (46.51% vs. 45.75%) when only the 2013 data is used in training.

**Table 11:** Impact of 2011 and 2013 data on GE test set.

Data Attribute	Recall	Precision	F-score
2011 data	35.60%	66.09%	46.27%
2013 data	35.90%	66.02%	46.51%

Based on the presented results, our extensions developed after BioNLP-ST 2013 contribute mostly to the event extraction precision. We investigated the possible reason for the recall loss by closely examining the process of the ERM-based optimization. We realized that the graph patterns derived from the shortest paths connecting event arguments are sometimes too compact, for instance the *Positive_regulation *pattern "BIO_Entity activities" and the *Gene_expression *pattern "presence of BIO_Entity". The information redundancy of these patterns is 0 because the participating biological entities are directly connected with the event triggers "activities" and "presence". However, due to the lack of a more detailed event context, such as a description on the kind of "activities" or an environment causing the "presence", while producing correct predictions, these patterns incur a much higher number of false positive events. Therefore, even though the subsequent removal of these patterns by the optimization module ensures the overall precision, it results in an inevitable recall decrease. Recovering the correct predictions from these patterns requires additional context information beyond the current graph representation. In this regard, supervised learning-based systems have demonstrated their ability in exploring a broader context in a sentence by taking advantage of all individual words and sequences of words [6,12,13].

### CG task

#### Datasets

The dataset of the CG task is based on an existing corpus composed of abstracts from the angiogenesis domain [37]. The CG task targeted a challenging group of 40 cancer biology related event types and involved 18 kinds of biological entities describing the development and progression of cancer [38,39]. Table 12 presents some statistics of the CG dataset.

**Table 12:** Statistics of BioNLP-ST 2013 CG dataset.

Attributes Counted	Training	Development	Test
Abstracts	300	100	200
Entities	10,935	3,634	6,955
Annotated events	8,803	2,915	5,972 (hidden)

#### CG results on test set in BioNLP-ST 2013

When generalizing our system to the CG event extraction task, we also incorporated the corresponding annotated data from the 2011 tasks into the training phase for pattern induction. Considering the GE task has been the signature task of the BioNLP shared task series since 2009, we focused our methodological extension attempts on the GE task dataset. However, neither attempt led to significant improvement on the GE task dataset. We consider that a real methodology improvement should be independent of datasets. Therefore, we only applied our base system [4] to the CG task dataset to demonstrate its generalization ability. We did not further explore the three newly proposed extensions on the CG dataset given their ineffectiveness on the GE task test data.

Our NCBI submission ranks 3*^rd ^*among 6 different participating teams of the CG task, achieving a F-score of 46.38% on the 200 test documents. The primary evaluation results of all participating teams are given in Table 13. The only two teams that participated in both GE and CG tasks are "TEES-2.1" and our team. The detailed results in terms of each of the 40 event types are provided on the official website of the CG task [39,40].

**Table 13:** Performance of all systems in 2013 CG task.

Team	Recall	Precision	F-score
TEES-2.1	48.76%	64.17%	55.41%
NaCTeM	48.83%	55.82%	52.09%
**NCBI**	38.28%	58.84%	46.38%
RelAgent	41.73%	49.58%	45.32%
UET-NII	19.66%	62.73%	29.94%
ISI	16.44%	47.83%	24.47%

The task organizers provided annotations for all biological entities but not for the optional "Site" argument occurred in events such as "Mutation", "Binding" and "Phosphorylation". Since entity recognition such as detecting "Site" entities is beyond the event extraction itself, we ignored the "Site" argument in our system. However, this leads to a problem in the evaluation that an event will be considered false positive if a "Site" argument is not identified although the other arguments are all correctly detected. Furthermore, the overall task evaluation considers the detection of modifications of the predicted events such as negation and speculation. These arguments are required by the secondary task of the CG task and appear in about 7.5% of the annotated test instances. Thus, missing these arguments in our results directly damages our final recall as we focused on the primary task only. We have requested the organizers to conduct an additional evaluation on core event extraction targets without optional arguments such as "Site" and arguments from the secondary task. More detailed analysis will be conducted on the results as soon as they become available.

## Conclusion and future work

In the BioNLP-ST 2013, we adapted our ASM-based system to the GE and CG event extraction tasks. We attempted to integrate semantics into the graph matching scheme using a distributional similarity model for more events. We also evaluated the event extraction impact of using paths of all possible lengths as key context dependencies beyond using only the shortest paths in our system. We achieved a 46.38% F-score in the CG task, ranking 3*^rd^*and a 48.93% F-score in the GE task, ranking 4*^th^*.

After the 2013 challenge, we further explored three other ways to extend our system. We redesigned our ASM algorithm by allowing nodes in pattern graphs to be skipped with penalty, learned a customized threshold for each pattern, and optimized the event pattern set following the empirical risk minimization principle. We demonstrated the impact of various system settings on the event extraction performance. Our extensions lead to a high 65% event extraction precision. However, due to a 1% recall decrease, we achieved a F-score identical to our original performance in the shared task.

In our future work, we are interested in investigating a more appropriate method to determine the set of non-essential nodes in pattern graphs. This will help the ASM algorithm determine the correct pattern nodes to skip, thus guiding the correct generalization of patterns. We also plan to integrate our graph pattern representation and the ASM subgraph distance with supervised learning algorithms to take advantage of their ability of exploring a much broader event context. While the integration of a distributional similarity model in our system did not lead to a performance improvement, in the future we intend to use the method introduced in [32] to address the antonym problem.

## Competing interests

The authors declare that they have no competing interests.

## Authors' contributions

HL conducted all the experiments and drafted the manuscript. KV participated in the project, contributed to the manuscript and provided valuable comments on the design of the experiments. DCC preprocessed all the data into the BioC compliant XML format. AM participated in the project and contributed to the software engineering of the event extraction system. WJW supervised the design of the project and contributed to the manuscript. All authors have read and approved this manuscript.
